# The mitochondrial genome of the chimpanzee louse, *Pediculus schaeffi*: insights into the process of mitochondrial genome fragmentation in the blood-sucking lice of great apes

**DOI:** 10.1186/s12864-015-1843-3

**Published:** 2015-09-03

**Authors:** Kate E. Herd, Stephen C. Barker, Renfu Shao

**Affiliations:** Department of Parasitology, School of Chemistry and Molecular Biosciences, The University of Queensland, Brisbane, QLD Australia; GeneCology Research Centre, Faculty of Science, Education and Engineering, University of the Sunshine Coast, Maroochydore, QLD Australia

**Keywords:** Chimpanzee louse, *Pediculus schaeffi*, Mitochondrial genome, Minichromosomes

## Abstract

**Background:**

Blood-sucking lice in the genera *Pediculus* and *Pthirus* are obligate ectoparasites of great apes. Unlike most bilateral animals, which have 37 mitochondrial (mt) genes on a single circular chromosome, the sucking lice of humans have extensively fragmented mt genomes. The head louse, *Pediculus capitis*, and the body louse, *Pe. humanus*, have their 37 mt genes on 20 minichromosomes. The pubic louse, *Pthirus pubis*, has its 34 mt genes known on 14 minichromosomes. To understand the process of mt genome fragmentation in the sucking lice of great apes, we sequenced the mt genome of the chimpanzee louse, *Pe. schaeffi*, and compared it with the three human lice.

**Results:**

We identified all of the 37 mt genes typical of bilateral animals in the chimpanzee louse; these genes are on 18 types of minichromosomes. Seventeen of the 18 minichromosomes of the chimpanzee louse have the same gene content and gene arrangement as their counterparts in the human head louse and the human body louse. However, five genes, *cob*, *trnS*_*1*_*, trnN*, *trnE* and *trnM*, which are on three minichromosomes in the human head louse and the human body louse, are together on one minichromosome in the chimpanzee louse.

**Conclusions:**

Using the human pubic louse, *Pt. pubis*, as an outgroup for comparison, we infer that a single minichromosome has fragmented into three in the lineage leading to the human head louse and the human body louse since this lineage diverged from the chimpanzee louse ~6 million years ago. Our results provide insights into the process of mt genome fragmentation in the sucking lice in a relatively fine evolutionary scale.

**Electronic supplementary material:**

The online version of this article (doi:10.1186/s12864-015-1843-3) contains supplementary material, which is available to authorized users.

## Background

The closest extant relatives of humans are chimpanzees (bonobos and common chimpanzees) and gorillas. Humans share a common ancestor with gorillas, *Gorilla gorilla* and *G. beringei*, ~7 million years ago (MYA) and share a common ancestor with chimpanzees, *Pan troglodytes* and *Pan paniscus*, ~6 MYA ([1, 2], but see [3–5] for variation of the divergence time estimates). Due to the close evolutionary relationship between humans and these great apes, their parasites are closely related too [6]. All great apes, with the exception of orangutans, *Pongo pygmaeus*, are parasitised by sucking lice (suborder Anoplura) of two genera, *Pediculus* and *Pthirus* [7, 8].

Humans are parasitised by three species of lice. *Pediculus capitis*, the head louse, lives on human head hair and is still prevalent in many countries, particularly in school-aged children. *Pe. humanus*, the human body louse, lives on human clothes and is thought to have evolved to exploit this new niche when humans began to wear clothing 100,000 to 200,000 years ago [9–11]. Although not as prevalent as the head lice, body lice are healthily more important as they are vectors for three pathogens that cause trench fever, recurrent fever and epidemic typhus in humans [12]. Humans also have the pubic louse, *Pthirus pubis*, which lives on the pubic hair and sometimes eyelashes [13]. The human head louse and the body louse share a genus exclusively with the chimpanzee louse, *Pe. schaeffi*, whereas the human pubic louse shares a genus exclusively with the gorilla louse, *Pt. gorilla* [7, 8, 14, 15].

The mitochondrial (mt) genomes of the three human lice have fragmented extensively. The typical mt genomes of bilateral animals have 37 genes on a single circular chromosome [16, 17]. The human head louse and the body louse, however, have their 37 mt genes on 20 types of minichromosomes [18, 19]. The 34 mt genes known of the human pubic louse are on 14 minichromosomes [19]. To understand the process of mt genome fragmentation in the sucking lice of great apes, we sequenced the mt genome of the chimpanzee louse, *Pe. schaeffi*, and compared it with those of the human lice. We found that the 37 mt genes of the chimpanzee louse are on 18 types of minichromosomes. We show that a single minichromosome has fragmented into three in the lineage leading to the human body louse and head louse since this lineage diverged from the chimpanzee louse ~6 million years ago. Our results provide insights into the process of mt genome fragmentation in the sucking lice in a relatively fine evolutionary scale.

## Methods

### Collection of lice, DNA extraction, mitochondrial genome amplification and sequencing

Chimpanzee lice, *Pe. schaeffi*, were collected from rescued wild chimpanzees by staff of the Tacugama Chimpanzee Sanctuary in Sierra Leone (sample No. B2148) and were preserved in 100 % ethanol. No animal ethical approval was required for research on parasitic lice including chimpanzee lice and human lice in Australia. Total cellular DNA was extracted from individual lice using DNeasy Blood and Tissue kit (QIAGEN). A 423-bp fragment of mt *cox1* gene and a 503-bp fragment of mt *rrnL* gene of the chimpanzee lice were amplified initially by PCR with primer pairs mtd7–mtd9a and mtd32m–mtd34Ph (Additional file 1). These primers target conserved sequence motifs in the mt genome of the chimpanzee lice. The *cox1* and *rrnL* fragments were sequenced directly with AB3730*xl* 96-capillary sequencers at the Australian Genome Research Facilities (AGRF).

Two pairs of chimpanzee louse specific primers, Pscox1Frev-Pscox1Rrev and P:sh16SF-16SrevP:schaeffi, were designed from *cox1* and *rrnL* (Additional file 1). PCRs with these chimpanzee louse specific primers amplified the complete *cox1* minichromosome (~3.3 kb, Additional file 2A) and *L*_*2(/1)*_*-rrnL* minichromosome (~3.0 kb, Additional file 2B) except a 203-bp and a 231-bp gap between the primers, respectively. PCR amplicons from these two minichromosomes were sequenced using a primer-walking strategy with AB3730*xl* 96-capillary platform at the AGRF (see Additional file 1 for primers used). Sequences of the non-coding regions of the *cox1* minichromosome and *L*_*2(/1)*_*-rrnL* minichromosome were obtained and aligned with ClustalX [20]. A forward primer (PsF) and a reverse primer (PsR) were designed from highly conserved non-coding sequences adjacent to the 5′-end and the 3′-end, respectively, of the coding regions (Additional files 1 and 3). The PCR with PsF and PsR produced a mixture of amplicons ranging from 200 to 1800 bp from the coding regions of the mt minichromosomes of the chimpanzee louse (Additional file 2C). Amplicons generated with PsF − PsR were sequenced with a Roche GS FLX platform at the AGRF. To obtain non-coding region sequences, full-length *trnK-nad4* minichromosome and *trnL-rrnS-trnC* minichromosome were also amplified by PCR with chimpanzee louse specific primers (Additional file 1), and were sequenced together with *cox1-*minichromosome amplicons using an Illumina Hiseq platform at the Beijing Genomics Institute (BGI).

Takara LA *Taq* was used in PCR following the manufacturer’s protocol. Each PCR (25 μL) contained 0.25 μL of LA *Taq*, 2.5 μL of 10× Buffer, 2.5 μL of MgCl_2_ (25 mM), 2.0–4.0 μL of dNTP mixture (2.5 mM each), 1.0 μL of forward primer (10 μM), 1.0 μL of reverse primer (10 μM), 1.0 μL of DNA template, and 12.75–14.75 μL of Milli-Q water. Thermal cycling conditions were 94 °C for 1 min; then 38 cycles of 94 °C for 30 s; 50–58 °C (depending on primers) for 30 s; 68 °C for 1–4 min (depending on amplicon size); and finally 68 °C for 2–8 min (depending on amplicon size). Negative controls were run with each PCR experiment to detect false positive amplicons and DNA contamination. PCR products were checked by agarose gel eletrophoresis (1 %); the sizes of PCR products were estimated by comparing with molecular markers. PCR products were purified using the Wizard® SV Gel and PCR Clean-up System (Promega).

### Assembly of Roche and Illumina sequence reads and mitochondrial genome annotation

Raw sequence-reads were assembled *de novo* using Geneious [21] with the parameters: 1) minimum overlap 150 bp (for Roche reads) and 70 bp (for Illumina reads); 2) minimum overlap identity 95 %; 3) maximum 10 % gaps per read; and 4) maximum gap size 10 bp. Protein-coding and rRNA genes were identified with BLAST searches (Basic Local Alignment Search Tool) of NCBI database [22] and verified by sequence alignment with their homologous mt genes of the human lice [18, 19]. tRNA genes were identified with tRNAscan-SE [23], ARWEN [24] and MITOS [25]. Shared identical sequences between genes were identified with Wordmatch [26]. Sequence alignment was made with Clustal X [20].

## Results and discussion

### The mitochondrial genome of the chimpanzee louse comprises 18 types of minichromosomes

**Fig. 1:**
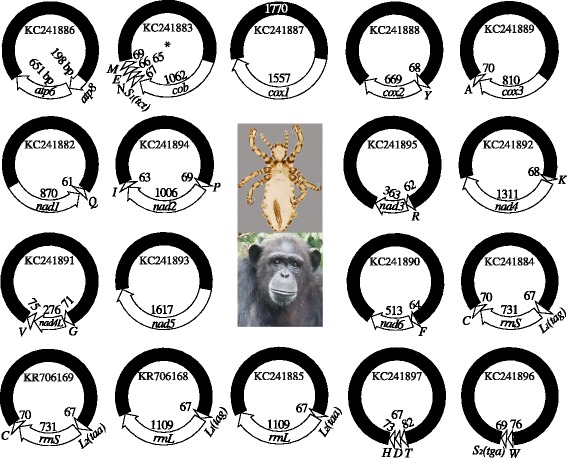
The mitochondrial minichromosomes of the chimpanzee louse, *Pediculus schaeffi*. Each minichromosome has a coding region with gene names, transcription orientation, and length indicated, and a NCR in black. The minichromosomes are in alphabetical order according to the names of their protein-coding and rRNA genes, followed by those with tRNA genes only. Protein-coding genes are abbreviated as *atp6* and *atp8* (for ATP synthase subunits 6 and 8), *cox1-3* (for cytochrome c oxidase subunits 1 to 3), *cob* (for cytochrome b), and *nad1-6* and *4 L* (for NADH dehydrogenase subunits 1 to 6 and 4 L). *rrnL* and *rrnS* are for large and small rRNA subunits. tRNA genes are shown with the single-letter abbreviations of their corresponding amino acids. Chimpanzee image: courtesy of the Tacugama Chimpanzee Sanctuary

We sequenced the amplicons generated with the primer pair, PsF–PsR, from the chimpanzee louse, *Pe. schaeffi*, and obtained 24,230 sequence-reads, which range from 150 to 602 bp long. We assembled these sequence-reads into contigs and identified all of the 37 mt genes typical of bilateral animals in the chimpanzee louse; these genes are on 18 types of minichromosomes (Fig. 1; Table 1). Each minichromosome has 1–5 genes and is 3–4 kb in size, with a coding region, 145 (for *trnW-trnS*_*2*_ minichromosome) to 1617 bp (for *nad5* minichromosome) in size, and a non-coding region (NCR). Seventeen of the 18 mt minichromosomes of the chimpanzee louse have their counterparts with the same gene content and gene arrangement in the human head louse, *Pe. capitis*, and the human body louse, *Pe. humanus* [18, 19]. Only *cob-trnS*_*1*_*-trnN-trnE-trnM* minichromosome of the chimpanzee louse is not seen in the human head louse and the body louse; the five genes on this minichromosome are on three minichromosomes in the two human lice.

We identified 21 of the 22 mt tRNA genes of the chimpanzee louse with tRNAscan-SE [23], ARWEN [24] and MITOS [25] on the basis of their inferred secondary structures and anticodon sequences (Additional file 4). We could not identify *trnD* with these programs. Instead, we used the *trnD* sequences of the human head louse and the body louse [19] to locate this gene in the chimpanzee louse. *trnD* of the chimpanzee louse is on a minichromosome with *trnT* and *trnH* and has 72 % sequence similarity to *trnD* of the human head louse and body louse (Additional file 4). Furthermore, the location of *trnD* of the chimpanzee louse relative to their neighbour genes is the same as that in the human head louse and the body louse (Fig. 1) [18, 19].

As in the human lice [19], *trnL*_*1*_*(tag)* and *trnL*_*2*_*(taa)* of the chimpanzee louse differ by only one nucleotide at the third anticodon position (Additional file 4). Our Roche deep sequencing revealed that both *trnL*_*1*_*(tag)* and *trnL*_*2*_*(taa)* were present before *rrnS* and *rrnL* in the chimpanzee louse: for *rrnS* the vast majority were *trnL*_*1*_ (94.5 %) whereas for *rrnL* the vast majority were *trnL*_*2*_ (97.3 %) (Fig. 1). The relative abundance between the two *trnL* genes in the four types of minichromosomes that contain *rrnS* and *rrnL* genes of the chimpanzee louse was consistent with that of the human head louse and the body louse we investigated (Additional file 5).

We sequenced the full-length NCR of *cox1* minichromosome with an Illumina Hiseq platform; we were unable, however, to sequence a gap downstream the GC-rich motif in the NCR of *trnK-nad4* minichromosome, nor *trnL-rrnS-trnC* minichromosome, although we used the same sequencing strategy. The NCR of *cox1* minichromosome is 1770 bp long with a GC-rich motif and an AT-rich motif at two ends that are highly conserved in other minichromosomes. There are two repeat units in the NCR of *cox1* minichromosome, 112 and 114 bp long respectively; these two units are 406 bp apart from one another and have 94 % similarity (Additional file 3).

### Recombination hot-spots revealed by shared identical sequences between mitochondrial genes in the chimpanzee louse

**Fig. 2:**
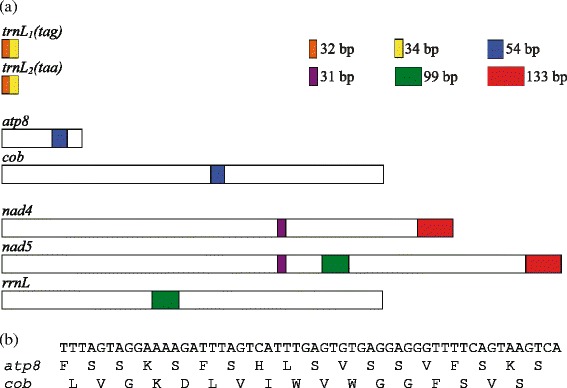
Recombination hot-spots in the mitochondrial genomes of the chimpanzee louse, *Pediculus schaeffi*, indicated by shared identical sequences between non-homologous genes. **a**: locations of shared identical sequences in genes. Shared identical sequences are highlighted in color with their length in bp. Genes are indicated with boxes from 5′ end to 3′ end. **b**: the 56-bp identical sequence shared between *atp8* and *cob* used different open reading frames

Seven stretches of identical nucleotide sequences, 31 to 133 bp long, were found between five pairs of mt genes in the chimpanzee louse (Table 2). As in the three human lice [19], *trnL*_*1*_ and *trnL*_*2*_ of the chimpanzee louse differ by only one nucleotide at the third anticodon position, and thus share two stretches of identical sequences, 32 and 34 bp respectively (Fig. 2a; Additional file 4). *nad5* and *rrnL* share 101-bp identical sequence in the chimpanzee louse; these two genes share 99-bp identical sequence in the human head louse and the human body louse, but not in the human pubic louse [19], nor in other blood-sucking lice [27]. *cox1* and *nad3* share 49 bp of identical sequence in the chimpanzee louse; these two genes, however, do not share longer-than-expected identical sequences in the human lice, nor in other sucking lice. *atp8* and *cob* share 26 bp of identical sequence in the human head and body lice but share 54 bp of identical sequence in the chimpanzee louse. *nad4* and *nad5* share two stretches of identical sequences, 31 and 133 bp long, in the chimpanzee louse; in the human head louse and body louse, these two genes share 30 and 127 bp identical sequences.

**Table 1:** Mitochondrial minichromosomes of the chimpanzee louse, *Pediculus schaeffi*, identified by Roche 454 sequencing

Minichromosome	Length of coding region (bp)	Number of Roche sequence-reads^a^
*atp*8-*atp6*	838	1372
*cob*-*trnS1*-*trnN*-*trnE*-*trnM*	1354	692
*cox1*	1557	764
*trnY*-*cox2*	737	688
*cox3- trnA*	884	1357
*nad1*-*trnQ*	964	1448
*trnP*-*nad2*-*trnI*	1138	553
*trnR*-*nad3*	425	3433
*trnK*-*nad4*	1379	336
*trnG*-*nad4L*-*trnV*	420	7038
*nad5*	1617	158
*trnF-nad6*	573	2149
*trnL*_*1(2)*_-*rrnS*-*trnC* ^b^	916	1628
*trnL*_*2(1)*_-*rrnL* ^c^	1224	1263
*trnW-trnS*_*2*_	145	135
*trnT*-*trnD*-*trnH*	233	1216

^a^The number of sequence-reads depended on the assembly parameters; the parameters used here were: minimum overlap 150 bp; minimum overlap identity 95 %; maximum 10 % gaps per read; and maximum gap size 10 bp

^b^Both *trnL*_*1*_ *(tag)* (94.5 %) and *trnL*_*2*_ *(taa)* (5.5 %) were found before *rrnS*; ^c^both *trnL*_*1*_ (2.7 %) and *trnL*_*2*_ (97.3 %) were found before *rrnL* (Additional file 5)

**Table 2:** The longest identical sequences shared by mitochondrial genes of the chimpanzee louse and the human lice, which have fragmented mitochondrial genomes, and six other species of animals, which have typical mitochondrial genomes

Pair of gene		The longest shared identical sequences (bp)									
		Ps	Pc	Ph	Pp	Bm	Cb	Hm	Dy	Ce	Hs
*trnL*_*1*_	*trnL*_*2*_	**32, 34**	**32, 33**	**32, 33**	**32, 35**	7	6	7	10	6	6
*trnG*	*trnR*	10	**14, 28**	**14, 28**	**26, 32**	5	6	7	6	8	6
*trnI*	*trnT*	7	6	6	**16**	6	5	7	7	9	6
*cox1*	*nad4L*	11	10	10	**29**	13	11	14	13	12	10
*nad5*	*rrnL*	**101**	**99**	**99**	10	12	14	13	15	16	10
*nad2*	*rrnL*	11	**26**	**26**	10	13	11	14	13	12	10
*cox1*	*nad3*	**49**	11	10	11	13	11	12	13	11	12
*atp8*	*cob*	**54**	**26**	**26**	9	10	11	11	12	N.A.	9
*nad4*	*nad5*	**31, 133**	**30, 130**	**30, 130**	N.A.	13	15	15	16	14	11

Shared identical sequences longer than expected by chance are in bold

Ps *Pediculus schaeffi* (chimpanzee louse), Pc *Pediculus capitis* (human head louse), Ph *Pediculus humanus* (human body louse), Pp *Pthirus pubis* (human pubic louse), Bm *Bothriometopus macrocnemis* (screamer louse), Cb *Campanulotes bidentatus* (pigeon louse), Hm *Heterodoxus macropus* (wallaby louse), Dy *Drosophila yakuba* (fruitfly), Ce *Caenorhabditis elegans* (roundworm), Hs *Homo sapiens* (human)

**Fig. 3:**
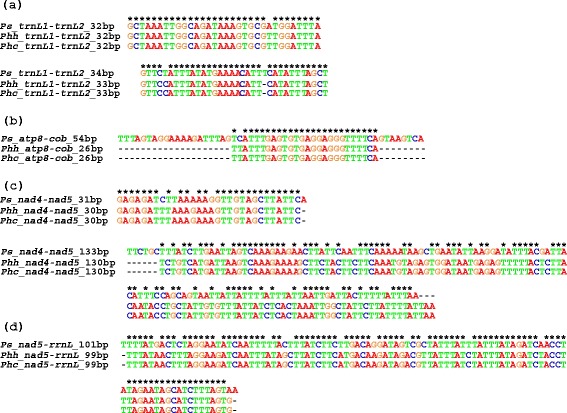
Alignment of identical sequences shared by non-homologous mitochondrial genes among the chimpanzee louse, *Pediculus schaeffi* (Ps)*,* the human body louse, *Pediculus humanus humanus* (Phh) and the human head louse, *Pediculus humanus capitis* (Phc). **a**: two stretches of identical sequences shared by *trnL*_*1*_ and *trnL*_*2*_. **b**: identical sequences shared by *atp8* and *cob*. **c**: two stretches of identical sequences shared by *nad4* and *nad5*. **d**: identical sequences shared by *nad5* and *rrnL*. Gaps generated by sequence alignment were indicated with “-”. Conserved sites were indicated with “*”

The 133-bp identical sequences shared between *nad4* and *nad5* in the chimp lice are at the 3′ ends of these two genes. The question arisen was whether or not these sequences were indeed part of *nad4* and *nad5*. We annotate these sequences as part of *nad4* and *nad5* on the basis that: 1) each of them is part of an open reading frame (ORF); 2) the two ORFs with these sequences included are in the usual, expected length of *nad4* and *nad5* genes of insects; 3) these sequences are only present in *nad4* and *nad5*, not in any other genes nor noncoding regions in the chimpanzee louse; and 4) these sequences have high similarity with their corresponding regions of *nad4* and *nad5* in the human lice (Fig. 3).

The longer-than-expected identical sequences shared between mt genes in the chimpanzee louse provide further evidence for recombination between mt minichromosomes in the blood-sucking lice [18, 19, 27–30]. Furthermore, with the only exception of the 49-bp identical sequence shared between *cox1* and *nad3*, which was found only in the chimpanzee louse (Table 2), all of the six other identical sequences shared by different genes in the chimpanzee louse were in similar length, had high similarity and were at the same gene locations as their counterparts in the human head louse and the body louse (Fig. 2a, Fig. 3). Given the chimpanzee louse had a common ancestor with the human head louse and the body louse ~6 MYA (see below), the conserved gene locations for the six shared identical sequences are clearly hot spots for homologous recombination between mt genes in the chimpanzee louse, the human head louse and the body louse (Fig. 3).

When two different genes, non-homologous to each other, share a stretch of identical sequence, it begs the question: from which of the two genes did the shared sequence originate? In the human head louse and the body louse, there is insufficient evidence for us to answer this question for any of the nine shared identical sequences [18, 19]. This is also the case for all of the shared identical sequences observed in the chimpanzee louse except for the 54-bp identical sequence shared between *atp8* and *cob* (Table 2). BLAST searches showed that this 54-bp sequence was from a domain of *cob* conserved among insects and thus had a *cob* origin; intriguingly, this 54-bp sequence used the second open reading frame in *cob* but used the first frame in *atp8* (Fig. 2b).

### Mitochondrial genome fragmentation in the blood-sucking lice of great apes

All great apes, with the exception of orangutans are parasitised by sucking lice of the genera *Pediculus* and *Pthrius* [7, 8]. The human head louse, *Pe. capitis*, and the human body louse, *Pe. humanus*, share a genus exclusively with the chimpanzee louse, *Pe. schaeffi*, whereas the human pubic louse, *Pt. pubis*, shares a genus exclusively with the gorilla louse, *Pt.* gorilla. Two hypotheses have been raised for the distribution of the sucking lice in the genera *Pediculus* and *Pthirus* among humans, chimpanzees and gorillas. The first hypothesis postulates a host-switch event of *Pthirus* lice from gorillas to humans, whereas the second hypothesis postulates a loss of *Pediculus* lice in gorillas and a loss of *Pthirus* lice in chimpanzees [14]. The first hypothesis is more parsimonious and assumes the coevolution of *Pediculus* lice with their hosts. Thus, the divergence between the human head louse and the human body louse on one hand, and the chimpanzee louse on the other hand, occurred at the time when their hosts, humans and chimpanzees, diverged ~6 MYA ([1, 2], but see [3–5] for variation of the divergence time estimates).

Most bilateral animals have 37 mt genes on a single circular chromosome, 15–20 kb in size [16, 17]. The human head louse and the human body louse, however, have their 37 mt genes on 20 types of minichromosomes [18, 19]. For the human pubic louse, the 34 mt genes identified are on 14 minichromosomes [19]. We show in the present study that the chimpanzee louse has two less mt minichromosomes than the head louse and the body louse, although it is closely related to the human lice and is in the same genus *Pediculus* (Fig. 1).

The data available to date indicated that mt genomes already became fragmented in the most recent common ancestor (MRCA) of all blood-sucking lice (suborder Apoplura), as all of the species from both major clades of the sucking lice that have been properly examined have fragmented mt genomes [18, 19, 27–30]. Minicircles with mt genes were reported in a *Damalinia* species (Trichodectidae, Ischnocera), indicating that fragmented mt genomes may also be present in chewing lice [31]. The extensive fragmentation of mt genomes in the human head louse and the body louse [18, 19] and in the chimpanzee louse, however, does not appear to be an ancestral condition for sucking lice but a derived condition for the human lice and the chimp louse in the genus *Pediculus* since all other sucking lice studied to date have less or much less fragmented mt genomes than the human lice and the chimpanzee louse [18, 19, 27–30].

**Fig. 4:**
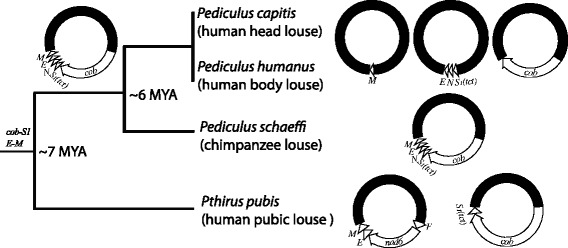
Fragmentation of *cob-trnS*_*1*_ *-trnN-trnE-trnM* minichromosome in the lineage leading to the human head louse, *Pediculus capitis*, and the human body louse, *Pe. humanus*. The split time, ~6 MYA, for the chimpanzee louse from the human head louse and the body louse was after Goodman (1999) [1] and Glazko and Nei (2003) [2] for the chimpanzee–human split time. The split time, ~7 MYA, for *Pthirus pubis* from the *Pediculus* species was also after Goodman (1999) [1] and Glazko and Nei (2003) [2] for the gorilla–(chimpanzee + human) split time

Among the 50 genera of sucking lice, *Pediculus* and *Pthirus* are most closely related to each other [7, 8, 14, 32, 33]. *Pthirus* species, thus, provide a proper outgroup for understanding mt genome fragmentation among the *Pediculus* species. Using the human pubic louse, *Pt. pubis*, as an outgroup for comparison (Fig. 4), we infer that: 1) the mt genome of the chimpanzee louse, which is less fragmented than that of the human head louse and the body louse, represents the ancestral condition for the genus *Pediculus* as the chimpanzee louse shares two gene arrangement characters, *cob-trnS*_*1*_ and *trnE-trnM*, with *Pt. pubis*; and 2) the *cob-trnS*_*1*_*-trnN-trnE-trnM* minichromosome fragmented into three: one with *cob*, one with *trnS*_*1*_*-trnN-trnE*, and the other with *trnM* in the lineage leading to the human head louse and the body louse after this lineage split from that leading to the chimpanzee louse. Furthermore, it is likely that the *cob-trnS*_*1*_*-trnN-trnE-trnM* minichromosome fragmented into two minichromosomes first: one with *cob* and the other with *trnS*_*1*_*-trnN-trnE-trnM*. The latter then fragmented further into another two, generating the three minichromosomes we see in the human head louse and the human body louse today (Figs. 1 and 4). If this was the case, it would indicate that the fragmentation of one minichromosome into two took approximately 3 MY.

## Conclusions

In conclusion, we sequenced the mt genome of the chimpanzee louse: the 37 mt genes typical of bilateral animals are on 18 minichromosomes in this louse. Seventeen of the 18 minichromosomes of the chimpanzee louse have the same gene content and gene arrangement as their counterparts in the human head louse and the body louse. Five genes, *cob*, *trnS*_*1*_*, trnN*, *trnE* and *trnM*, which are on three minichromosomes in the human head louse and the body louse, are on one minichromosome in the chimpanzee louse. Using the human pubic louse as an outgoup, we infer that a single minichromosome has fragmented into three in the lineage leading to the human head louse and the body louse since this lineage diverged from that leading to the chimpanzee louse ~6 MYA when their hosts diverged. Our results provided insights into the process of mt genome fragmentation in the sucking lice in a relatively fine evolutionary scale.

### Availability of supporting data

The nucleotide sequences of the mt genome of the chimpanzee louse supporting the results of this article have been deposited in GenBank (accession numbers KC241882–KC241897 and KR706168–KR706169). The mt genomes of the human body louse, head louse and pubic louse have been published previously (GenBank accession numbers: EU219983–EU219995, FJ499473–FJ499490, FJ514591–FJ514599, HM241895–HM241898, and JX080388– JX080407) [18, 19].

## Additional files

Additional file 1:
**Primers used to amplify and sequence the mitochondrial genome of the chimpanzee louse,** ***Pediculus schaeffi*** **.** (DOC 101 kb) Additional file 2:
**PCR amplicons from the mitochondrial genomes of the chimpanzee louse,** ***Pediculus schaeffi.*** (DOC 105 kb) Additional file 3:
**Alignment of the non-coding region sequences of the** ***cox1,*** ***trnK-nad4, trnL-rrnS-C*** **and** ***trnL-rrnL*** **minichromosomes of the chimpanzee louse,** ***Pediculus schaeffi.*** (DOC 108 kb) Additional file 4:
**The putative secondary structures of the 22 mitochondrial tRNAs of the chimpanzee louse,** ***Pediculus schaeffi.*** (DOC 89 kb) Additional file 5:
**The relative abundance between** ***trnL***_***1***_ ***(tag)*** **and** ***trnL***_***2***_ ***(taa)*** **genes in the four minichromosomes that contain** ***rrnS*** **and** ***rrnL*** **genes of the chimpanzee louse,** ***Pediculus schaeffi*** **, the human head louse,** ***Pe. capitis*** **, and the human body louse,** ***Pe. humanus*** **, revealed by Roche 454 sequencing.** (DOC 101 kb)

## Abbreviations

AGRF: Australian genome research facilities
*atp6* and *atp8*: Genes for ATP synthase subunits 6 and 8
bp: Base pair
*cob*: Gene for cytochrome b
*cox1, cox2* and *cox3*: Genes for cytochrome *c* oxidase subunits 1, 2 and 3
DNA: Deoxyribonucleic acid
kb: Kilo base pair
μl: Microliter
μM: Micromolar
min: Minute
MRCA: Most recent common ancestor
mt: Mitochondrial
MYA: Million years ago
*nad1, nad2*, *nad3*, *nad4*, *nad4L*, *nad5* and *nad6*: Mitochondrial genes for NADH dehydrogenase subunits 1–6 and 4 L
ORF: Open reading frame
PCR: Polymerase chain reaction
RNA: Ribonucleic acid
rRNA: Ribosomal RNA
*rrnS* and *rrnL*: Genes for small and large subunits of ribosomal RNA
sec: Second
T: Thymine
tRNA: Transfer RNA
tRNA: Transfer RNA
*trnA* or *A*: tRNA gene for alanine
*trnC* or *C*: tRNA gene for cysteine
*trnD* or *D*: tRNA gene for aspartic acid
*trnE* or *E*: tRNA gene for glutamic acid
*trnF* or *F*: tRNA gene for phenylalanine
*trnG* or *G*: tRNA gene for glycine
*trnH* or *H*: tRNA gene for histidine
*trnI* or *I*: tRNA gene for isoleucine
*trnK* or *K*: tRNA gene for lysine
*trnL*_*1*_ or *L*_*1*_: tRNA gene for leucine (anticodon NAG)
*trnL*_*2*_ or L_2_: tRNA gene for leucine (anticodon YAA)
*trnM* or *M*: tRNA gene for methionine
*trnN* or *N*: tRNA gene for asparagine
*trnP* or *P*: tRNA gene for proline
*trnQ* or *Q*: tRNA gene for glutamine
*trnR* or *R*: tRNA gene for arginine
*trnS1* or *S*_*1*_: tRNA gene for serine (anticodon NCU)
*trnS*_*2*_ or *S*_*2*_: tRNA gene for serine (anticodon NGA)
*trnT* or *T*: tRNA gene for threonine
*trnV* or *V*: tRNA gene for valine
*trnW* or *W*: tRNA gene for tryptophan
*trnY* or *Y*: tRNA gene for tyrosine
A: Adenine
G: Guanine
C: Cytosine
T: Thymine
U: Uracil
