# Supplementary material for: The mitochondrial genome of the chimpanzee louse, Pediculus schaeffi: insights into the process of mitochondrial genome fragmentation in the blood-sucking lice of great apes
Source: BMC Genomics. 2015 Sep 3;16(1):661. doi: 10.1186/s12864-015-1843-3 (PMC4557858; doi:10.1186/s12864-015-1843-3)
Supplement: Supplementary file 1 [file 12864_2015_1843_MOESM1_ESM.doc]

Additional file 1. Primers used to amplify and sequence the mitochondrial genome of the chimpanzee louse, *Pediculus schaeffi*

Target gene or region	Primer	Sequence (5' to 3')
cox1	mtd7	GGATCACCTGATATAGCATTCCC
cox1	mtd9a	CCAGGAAGAATAAGAATATAAACTTC
cox1	Pscox1Frev	CCTACACCGCCTTGAATTAAAGATCTTCT
cox1	Pscox1Rrev	CAGCGTTCTTACTACTTCTTTCTCTACCT
cox1	Psco1LNRF1	ACTCCTAGATTTATCATTAAGAGTGCGGT
cox1	Psco1LNRR2	GAGGTTTTGGAGTATTAACGGTCTTAATG
cox1	Pshco1Rseq	TGAATTAGATAGCACAACACCTGTAAGAC
cox1	Pshco1Fseq	TTCATGATACTTACTATGTTGTAGCTCAT
rrnL	mtd32m	ACGCCGGTCTGAACTCAAATCATGTAA
rrnL	mtd34Ph	TTTAGCCGGACTGTTTAATAAAAACAT
rrnL	P:sh16SF	ACTGCAGCTGTTAATTTAAACACTG
rrnL	16SrevP:schaeffi	ACTTTAAAGACTCAATTGTCACTTTATTG
Non-coding region	USRB2148	TCTCAACGGTTGAACCAAATGGCTACCCA
Non-coding region	PsF	GTACGGTTCAAACTCATTTCGAGTCAGCT
Non-coding region	PsR	AAGAGGTTTTGGAGTATTAACGG
nad4	Pshn4F	ATCGGTTGCGCTAGGTCGTTTTTTAGCCTT
nad4	Pshn4R	TCAACCACCTAAAGGAGACGGGTGTAGAGC
rrnS	PshrSF	ATAATGTGCCAGCACTAGCGGTCATACATT
rrnS	PshrSR	TTCTCAGGCCTTAATAATAAAACTAAGCTT
