# Supplementary material for: The mitochondrial genome of the chimpanzee louse, Pediculus schaeffi: insights into the process of mitochondrial genome fragmentation in the blood-sucking lice of great apes
Source: BMC Genomics. 2015 Sep 3;16(1):661. doi: 10.1186/s12864-015-1843-3 (PMC4557858; doi:10.1186/s12864-015-1843-3)
Supplement: Supplementary file 2 [file 12864_2015_1843_MOESM2_ESM.doc]

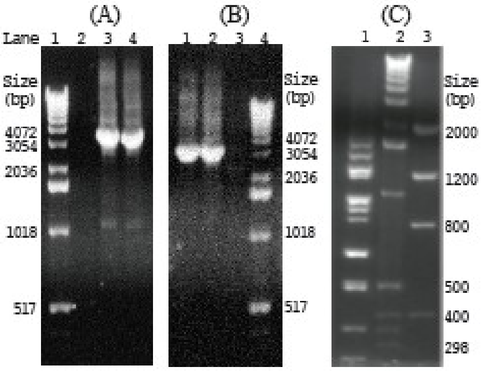

Additional file 2: PCR amplicons from the mitochondrial genomes of the chimpanzee louse, *Pediculus schaeffi*. (A) Lane 1: Molecular Weight Marker X (MWMX, Roche); lane 2: empty; lanes 3 and 4: amplicons generated with primer pair Pscox1Frev and Pscox1Rrev. (B) Lanes 1 and 2: amplicons generated with and P:sh16SF and 16SrevP:schaeffi; lane 3: empty; lane 4: MWMX. (C) Lane 1: amplicons generated with PsF and PsR; lane 2: MWMX; lane 3: Low DNA Mass Ladder (Invitrogen).
