# Supplementary material for: The mitochondrial genome of the chimpanzee louse, Pediculus schaeffi: insights into the process of mitochondrial genome fragmentation in the blood-sucking lice of great apes
Source: BMC Genomics. 2015 Sep 3;16(1):661. doi: 10.1186/s12864-015-1843-3 (PMC4557858; doi:10.1186/s12864-015-1843-3)
Supplement: Supplementary file 3 [file 12864_2015_1843_MOESM3_ESM.doc]

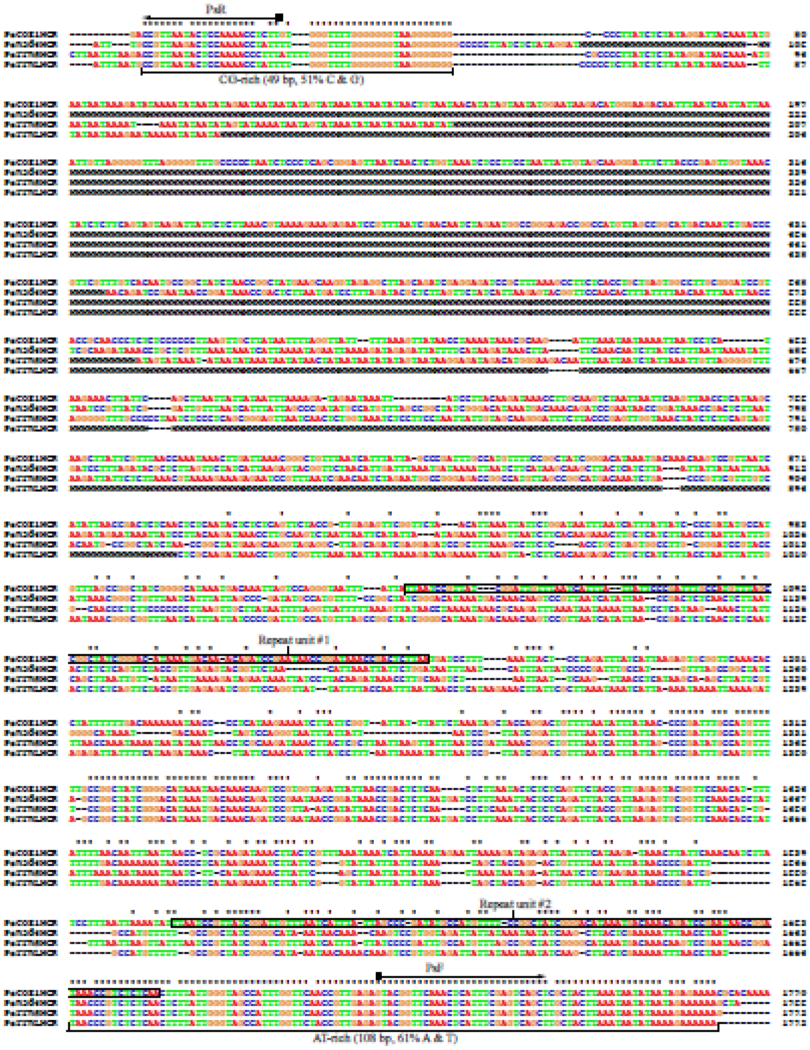

Additional file 3: Alignment of the non-coding region sequences of the *cox1*, *trnK-nad4*, *trnL-rrnS-C* and *trnL-rrnL* minichromosomes of the chimpanzee louse, *Pediculus schaeffi*. The unsequenced gaps downstream the GC-rich motif are indicated with strings of "N". The gaps generated by sequence alignment were indicated with "-". Conserved sites are indicated with "*". PsF and PsR are PCR primers use to amplify the coding regions shown in Figure 1.
