# Supplementary material for: The mitochondrial genome of the chimpanzee louse, Pediculus schaeffi: insights into the process of mitochondrial genome fragmentation in the blood-sucking lice of great apes
Source: BMC Genomics. 2015 Sep 3;16(1):661. doi: 10.1186/s12864-015-1843-3 (PMC4557858; doi:10.1186/s12864-015-1843-3)
Supplement: Supplementary file 4 [file 12864_2015_1843_MOESM4_ESM.doc]

Additional file 4. The relative abundance between *trnL₁(tag)* and *trnL₂(taa)* genes in the four minichromosomes that contain *rrnS* and *rrnL* genes of the chimpanzee louse, *Pediculus schaeffi*, the human head louse, *Pe. capitis*, and the human body louse, *Pe. humanus*, revealed by Roche 454 sequencing

**Sample number**	**Species**	***trnL-rrnS-trnC* minichromosome**		***trnL-rrnL* minichromosome**	
		trnL$_1$(tag)	***trnL$_2$(taa)***	***trnL$_1$(tag)***	***trnL$_2$(taa)***
B2148	*Pediculus schaeffi*	409/433=94.5%	24/433=5.5%	8/298=2.7%	290/298=97.3%
B1597A	*Pediculus capitis*	166/178=93.3%	12/178=6.7%	20/46=43.5%	26/46=56.5%
B1592B	*Pediculus humanus*	236/297=79.5%	61/297=20.5%	18/84=21.4%	66/84=78.6%
