# Supplementary material for: The mitochondrial genome of the chimpanzee louse, Pediculus schaeffi: insights into the process of mitochondrial genome fragmentation in the blood-sucking lice of great apes
Source: BMC Genomics. 2015 Sep 3;16(1):661. doi: 10.1186/s12864-015-1843-3 (PMC4557858; doi:10.1186/s12864-015-1843-3)
Supplement: Supplementary file 5 [file 12864_2015_1843_MOESM5_ESM.doc]

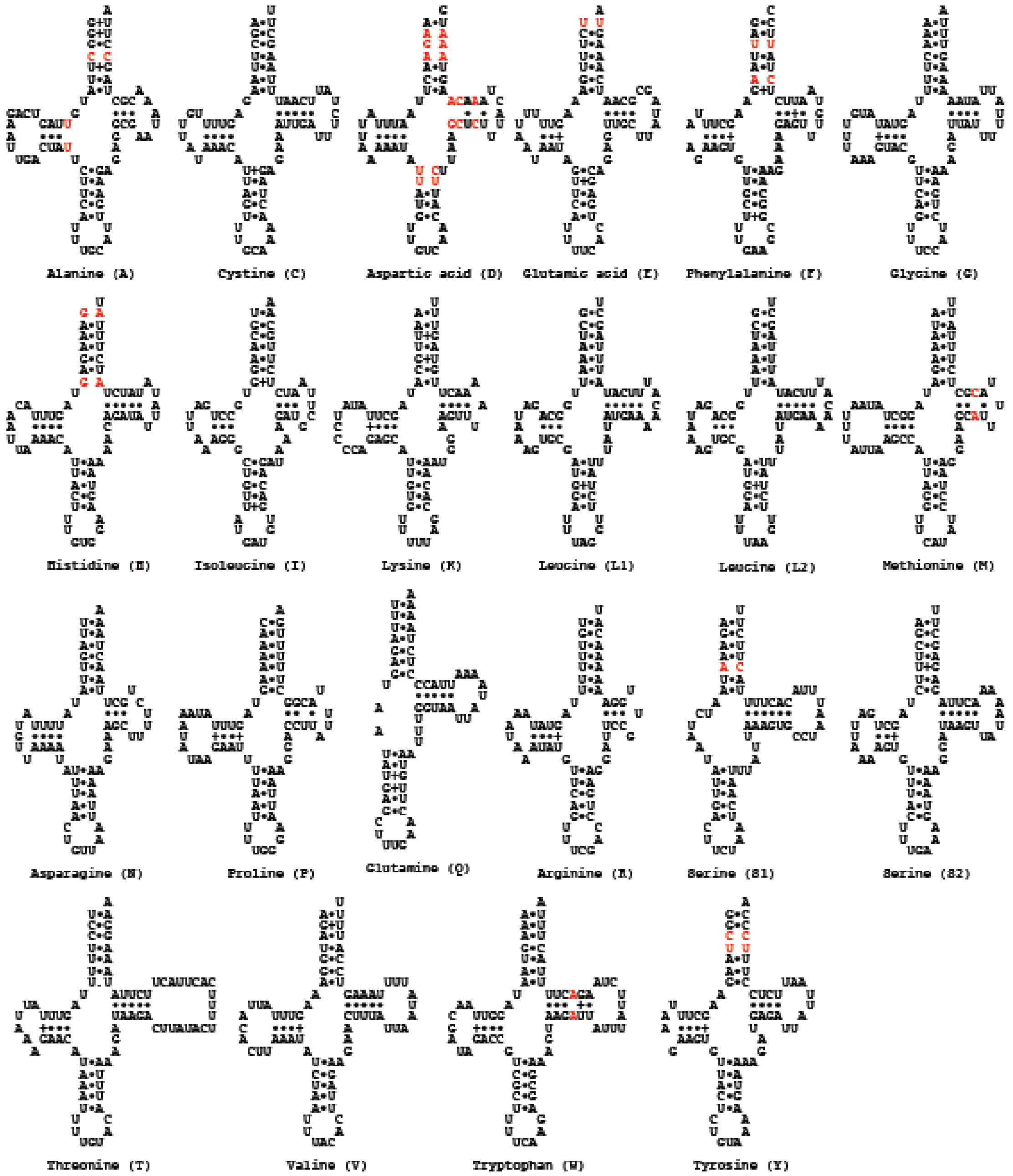

Additional file 5: The putative secondary structures of the 22 mitochondrial tRNAs of the chimpanzee louse, *Pediculus schaeffi*. Mismatches are in red. Conserved nucleotides in the tRNAs for aspartic acid and phenylalanine between the chimpanzee louse and the human head louse and the body louse are in bold. The identical nucleotides between the two tRNAs for leucine are also in bold.
